# Is the development of renewable energy projects compatible with rural communities? The case of Eastern China

**DOI:** 10.3389/fpubh.2026.1789344

**Published:** 2026-05-15

**Authors:** Zhuxiang Liu, Ajiang Chen, Yijun Liu

**Affiliations:** 1 Department of Sociology, Hohai University, Nanjing, China; 2 Research Center for Environment and Society, Hohai University, Nanjing, China; 3 Research Center for Green Development of Taihu Lake Basin, Hohai University, Nanjing, China

**Keywords:** Eastern China, energy transition, landscape compatibility, photovoltaic and wind energy, rural communities, social compatibility

## Abstract

**Introduction:**

The development of renewable energy projects has become a key strategy for addressing global climate change. From the perspective of social compatibility, this article examines the suitability of two types of renewable energy projects, photovoltaic and wind energy, for the local area.

**Methods:**

A mixed-methods approach combining interviews and questionnaires was used to obtain the attitudes of local residents, with the sample consisting of 96 respondents from four surrounding communities and villages.

**Results:**

The results show that the introduction of renewable energy projects has caused important changes to the environment, resource utilization, and landscape. The questionnaire results support the main viewpoints from the qualitative research.

**Conclusion:**

To improve social compatibility, targeted policy strategies are needed, including participatory planning that incorporates community views, support for local livelihood transitions, and differentiated strategies for photovoltaic and wind energy projects.

## 1 Introduction

Traditional fossil fuels produce large amounts of carbon dioxide, contributing to global climate change (1). This has become a significant issue of international concern (2). The development of clean energy effectively reduces carbon emissions and mitigates climate change (3, 4). In recent years, the Chinese government has actively promoted the development of renewable energy by advancing the construction of clean energy projects and promoting the transformation of the energy production structure into a green and low-carbon model. In particular, developing and utilizing clean energy sources such as wind and solar energy are effective means of optimizing the energy structure and improving energy efficiency (5).

In China’s eastern provinces, the development of renewable energy projects, particularly photovoltaic and wind power, has accelerated rapidly in recent years. However, these projects are often sited in rural and periurban areas where local communities depend on natural resources for their livelihoods. This geographic concentration raises an important question: Are renewable energy projects socially compatible with the rural communities that host them?

In the context of global climate change, although renewable energy has resulted in numerous environmental, economic, and social benefits, some social challenges in the energy transition have become increasingly apparent. Promoting renewable energy production is a process of social change in current society (6). Baker et al. (7) contend that the energy transition requires transformative changes to the energy system and involves a series of complex issues, such as the authority of regulatory agencies, market structure, and wealth distribution (8). The energy transition also involves addressing social and environmental challenges such as equity, justice, and sustainability (9). This paper focuses on the importance of a just transition in the development of renewable energy.

The development of renewable energy projects is not only the goal of decision makers but is also influenced by the opinions of local residents. Within the framework of environmental and energy justice, the views of residents living near renewable energy project sites, as stakeholders, cannot be ignored. Kalkbrenner and Roosen (10) point out that citizen participation can be an essential tool in energy transition at the local level. Although the municipal government has the decision-making power over the location of renewable energy, strong social resistance can have a negative impact on the low-carbon transition (11). Knudsen et al. (12) also examined the role of public participation in the decision-making processes of specific transmission line projects and investigated the views of local residents. These studies have provided important insights into the social aspects of the energy transition; however, overall they have focused less on systematically analyzing the interactions between projects and residents, especially with regard to the local communities that may be directly affected. Therefore, social compatibility provides a suitable analytical perspective, and it is necessary to systematically explore the compatibility between the development of renewable energy projects and local communities.

This study investigates whether the development of photovoltaic and wind energy projects is socially compatible with rural communities in Eastern China. Specifically, it addresses two research questions:

(1) What changes have renewable energy projects brought to local communities in terms of environment, resource utilization, and landscape?
(2) To what extent are photovoltaic and wind energy projects compatible with local communities across these three dimensions?

To answer these questions, this study adopts a mixed-methods approach, combining semistructured interviews and a questionnaire survey. The remainder of this paper is structured as follows. Section 2 reviews the relevant literature and presents the analytical framework of this study. Section 3 describes the study setting, participant recruitment, and data analysis methods. Section 4 presents the findings from the interviews and survey. Section 5 discusses the implications of the findings in relation to the existing literature. Section 5.1 concludes with policy recommendations and study limitations.

This study advances the existing literature in three key ways. First, it makes an empirical contribution by providing primary data on the social compatibility of photovoltaic and wind energy projects in rural Eastern China, a region underrepresented in current research. Second, methodologically, it adopts a mixed-methods approach, which enables triangulation of qualitative and quantitative findings and offers a more comprehensive assessment than single-method studies. Third, its policy value lies in identifying specific sources of incompatibility (e.g., landscape changes and resource utilization conflicts) and proposing corresponding adaptation strategies.

## 2 Literature review and conceptual framework

### 2.1 Literature review

#### 2.1.1 From adaptability analysis to social compatibility: concepts and their sources

The term “adaptation” originates from biology and describes how organisms adapt to environmental conditions and develop certain traits in the competition for survival. Generally, “adaptation” means being suitable for specific conditions or needs (13). In social science research, adaptation is inseparable from social development; thus, the concept of social adaptation has emerged. Social adaptation was first proposed by Herbert Spencer, who used Darwin’s theory of evolution to explain social phenomena and contended that society can be compared with biological organisms. He argued that the relationship between society and its members is like the relationship between biological individuals and cells. Therefore, in the process of interaction with the social environment, individuals strive to establish and maintain a harmonious and balanced relationship with it (14). When the social environment changes, the process by which individuals change their concepts and behaviors to adapt is called social adaptation.

In the field of project social evaluation, assessing social adaptability is one of the key criteria. It involves studying and predicting whether the local social and cultural environment can accept and support the existence of a project and its sustainable development, as well as examining the mutual adaptive relationship between the project and the local social environment (15).

Compatibility assessments are primarily used to explore the relationship between two or more elements that mutually influence each other and develop together (16). Compatibility studies mainly focus on the social evaluation of large-scale projects, such as offshore wind power projects, public housing, and highway construction. In addition, many studies have been conducted on the compatibility between traditional villages and the development of scenic areas and tourist attractions (17, 18).

In addition to developing a model or theory with a particular explanatory scope, a compatibility assessment must also establish a specific evaluation index for operationalization. The concept of compatibility reflects the fact that humans use certain ecosystem services while simultaneously making use of other service functions (19). Liu (19) developed an ecological compatibility model to access the ecological impact of port construction on wetland ecosystems, covering six aspects: external pressure, ecological service functions, landscape evaluation, ecological health, regional carrying capacity evaluation, and ecosystem risk evaluation. Existing studies have defined ecological compatibility as a state in which, over a certain period and within a relatively stable external environment, the structure and function of the ecosystem are in a state of dynamic balance characterized by high adaptability and coordination, with the entire system demonstrating good resistance and resilience. It has strong production, environmental service, landscape, and social and cultural functions (20). However, it is mainly implemented at the technical feasibility level, and there is a lack of research on the social level.

#### 2.1.2 Research on social acceptance in the renewable energy transition

Social acceptance is closely related to concepts such as social evaluation and social compatibility. In social science research, a project’s degree of compatibility also reflects social acceptance at the micro level, such as among resident groups and communities. For example, research has shown that a lack of social acceptance hinders the adoption of wind energy and jeopardizes action against global warming and climate change. The participation of the concerned residents and the municipality is important to overcome acceptance barriers (44). Due to the lack of institutionalized public participation channels, ordinary people are in a weak position when emission reduction policies are implemented, and their interests and concerns cannot be effectively expressed. At the same time, powerful groups dominate the policy formulation process (22).

#### 2.1.3 Economic, environmental, and social impacts of renewable energy development

Numerous existing studies have focused on the ecological and social issues, and related aspects, arising from the energy transition (2, 21, 23–28, 45); however, they have all focused on a specific aspect. Research on social compatibility is still insufficient, especially the lack of in-depth analyses from a social compatibility perspective exploring the development of renewable energy projects and their social impacts. Significant challenges remain in achieving a harmonious balance between the profitability of clean energy projects, sustainable community development, and climate change mitigation.

### 2.2 Theoretical and conceptual framework

Environmental justice includes the recognition of environmental justice communities, meaningful participation in environmental decision making, and the fair distribution of environmental burdens and benefits, with an emphasis on how these factors impact individual and communal capabilities and functioning (29). Environmental justice theory has also been applied to the process of energy transition, giving rise to the concepts of “climate justice” (30) and “energy justice” (31). The issue of social compatibility in the development of renewable energy projects essentially reflects a form of environmental injustice.

Social compatibility refers to the degree to which a new development policy, technology, or project is harmoniously integrated into the existing social context of a community or society. Ensuring that development or technology projects, such as renewable energy initiatives, have social compatibility is essential for securing local public support and making sure local communities benefit from these projects. This article develops an analytical framework for evaluating project–resident compatibility from three perspectives: environment, resource utilization, and landscape.

**Figure 1:**
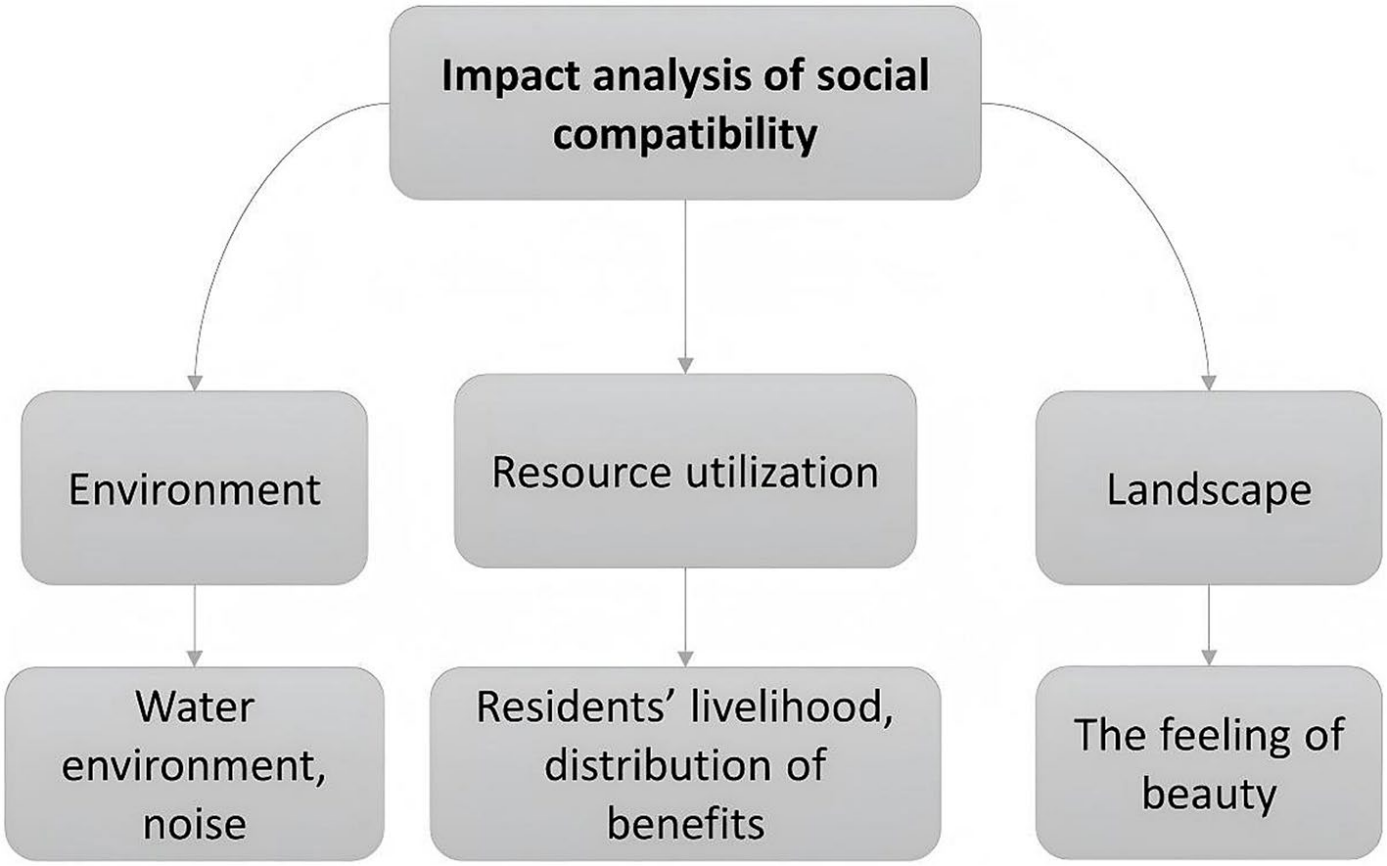
Framework diagram of the social compatibility impact analysis of renewable energy projects.

First, the mutual compatibility of the environment is examined by assessing the natural and living environment, primarily focusing on changes in the water environment and noise levels caused by the project, as well as residents’ degree of adaptation to the environment. Second, the mutual compatibility of resource utilization focuses on the changes in local livelihoods caused by the project and whether the distribution of benefits is appropriate. Third, landscape compatibility concerns the changes induced by the project to the natural landscape and the residents’ acceptance of the new landscape (see Figure 1).

## 3 Materials and methods

### 3.1 The development of renewable energy in China

During China’s green energy transition, the photovoltaic and wind energy industries have experienced rapid development. Since 2010, the installed capacity of photovoltaic and wind energy has maintained a steady annual growth, with particularly significant expansion over the past decade (48).

**Figure 2:**
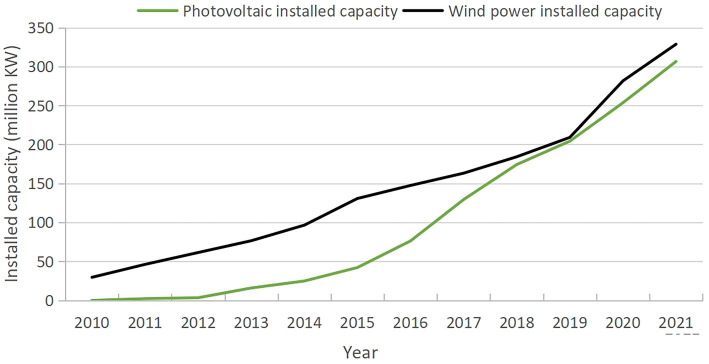
Installed photovoltaic and wind power generation capacity in China from 2010 to 2021. Source: NBSC (2022).

As can be seen from Figure 2, China’s photovoltaic installed capacity was only 200,000 kilowatts (0.2% of the national total) in 2011 but surged to 307 million kilowatts by 2021, accounting for 12.9% of the country’s total installed power generation capacity. Similarly, wind energy installed capacity increased from 46 million kilowatts (4.4% of the national total) in 2011 to 329 million kilowatts in 2021, representing 13.8% of the national installed capacity.

In terms of power generation, photovoltaic power output was 600 million kilowatt-hours in 2011, still at a nascent stage and accounting for a negligible share of national total power generation, while wind energy generation reached 74.1 billion kilowatt-hours (1.6% of the national total). By 2021, photovoltaic power generation had risen to 327 billion kilowatt-hours (3.9% of the national total), and wind energy generation had increased to 655.8 billion kilowatt-hours (7.8% of the national total).

The rapid expansion of the renewable energy industry has driven the widespread deployment of photovoltaic and wind energy projects across China, many of which are located in rural and periurban areas. This development provides the basis for the empirical investigation of project–community social compatibility presented in the following section.

### 3.2 Case study and context

**Figure 3:**
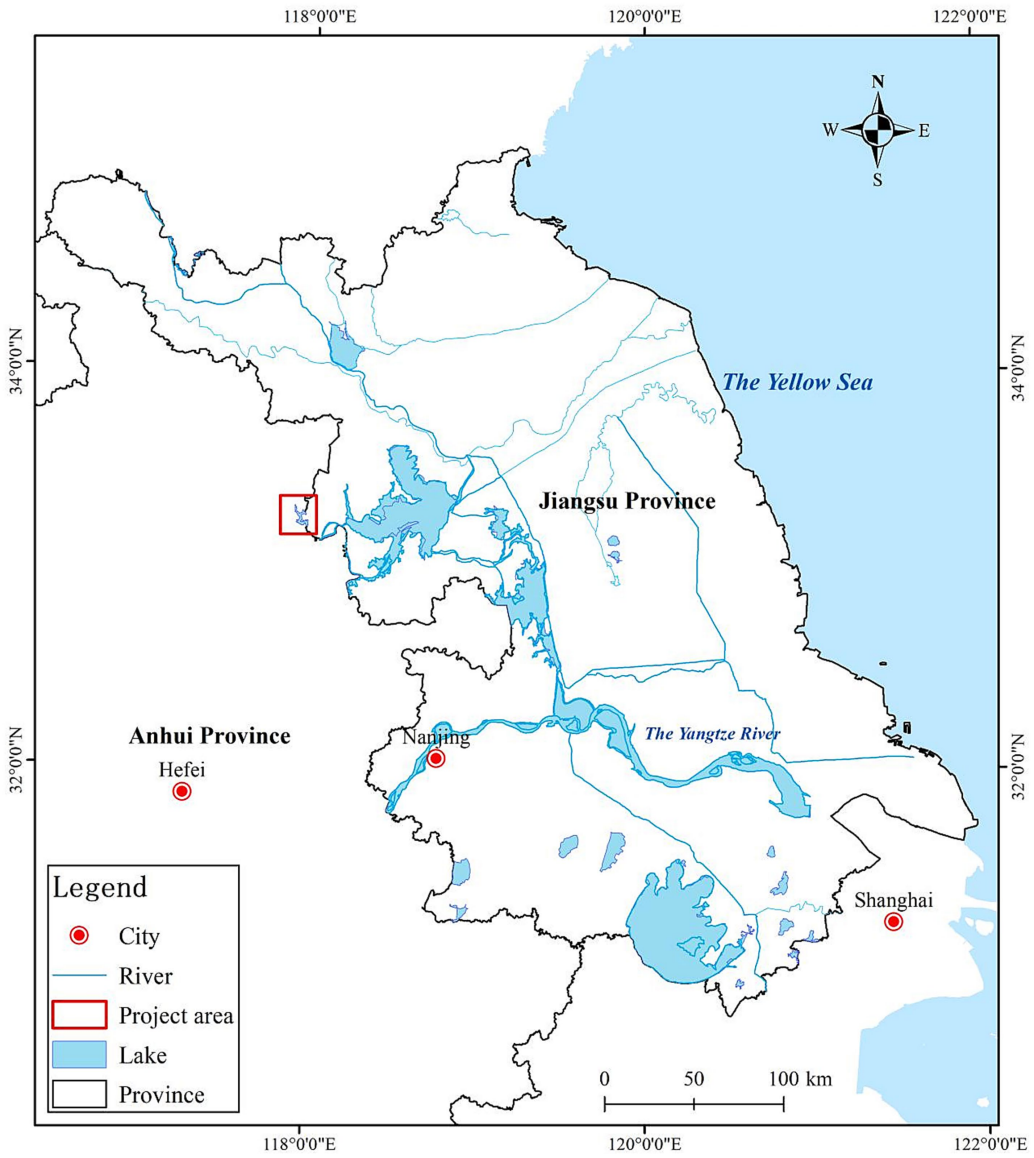
Location of Lan Lake on a map of Jiangsu Province.

Based on the research questions introduced above, this section describes the study setting in which the renewable energy projects are located. The research was conducted in Jiangsu Province, located along China’s eastern region. Within this province, the study focuses on a freshwater lake referred to here as Lan Lake (a pseudonym), situated in the western part of Jiangsu near the border with Anhui Province (see Figure 3). The lake’s eastern shoreline and the surrounding lowlands fall within Lan Township, where both photovoltaic and wind energy projects have been developed.

This location was selected for two main reasons. First, it offers a rare opportunity to compare photovoltaic and wind energy projects within the same geographic area, since both technologies have been installed in close proximity. Second, both projects had been operational for at least 2 years by the time of data collection, the photovoltaic project since 2019 and the wind power project since 2021, giving residents sufficient time to form stable opinions about their presence and impacts. To protect the identities of the research site and the individuals who participated in this study, the names of Lan Township and Lan Lake have been anonymized, and the names of enterprises, communities, and villages are represented by corresponding letters.

The photovoltaic project on Lan Lake is a piling-type water-based installation, meaning that the panels are supported by piles driven into the lakebed rather than floating on the surface. The project covers approximately 7.33 square kilometers of the lake’s surface, of which one enterprise, referred to as Enterprise W, occupies about 2.2 square kilometers. The total installed capacity of the photovoltaic project is roughly 500 megawatts. Although the primary purpose of the project is electricity generation, it also includes some aquaculture activity beneath the panels. The project began operating in 2019, and its development has been intensive: the photovoltaic installation occupies about one-third of Lan Lake’s total water area.

In addition to the photovoltaic project, a wind power project was completed in 2021, with approximately 25 wind turbines scattered across several communities and villages along the eastern shore of Lan Lake. These turbines are located in close proximity to residential areas, which has raised concerns among local residents about noise impact.

**Figure 4:**
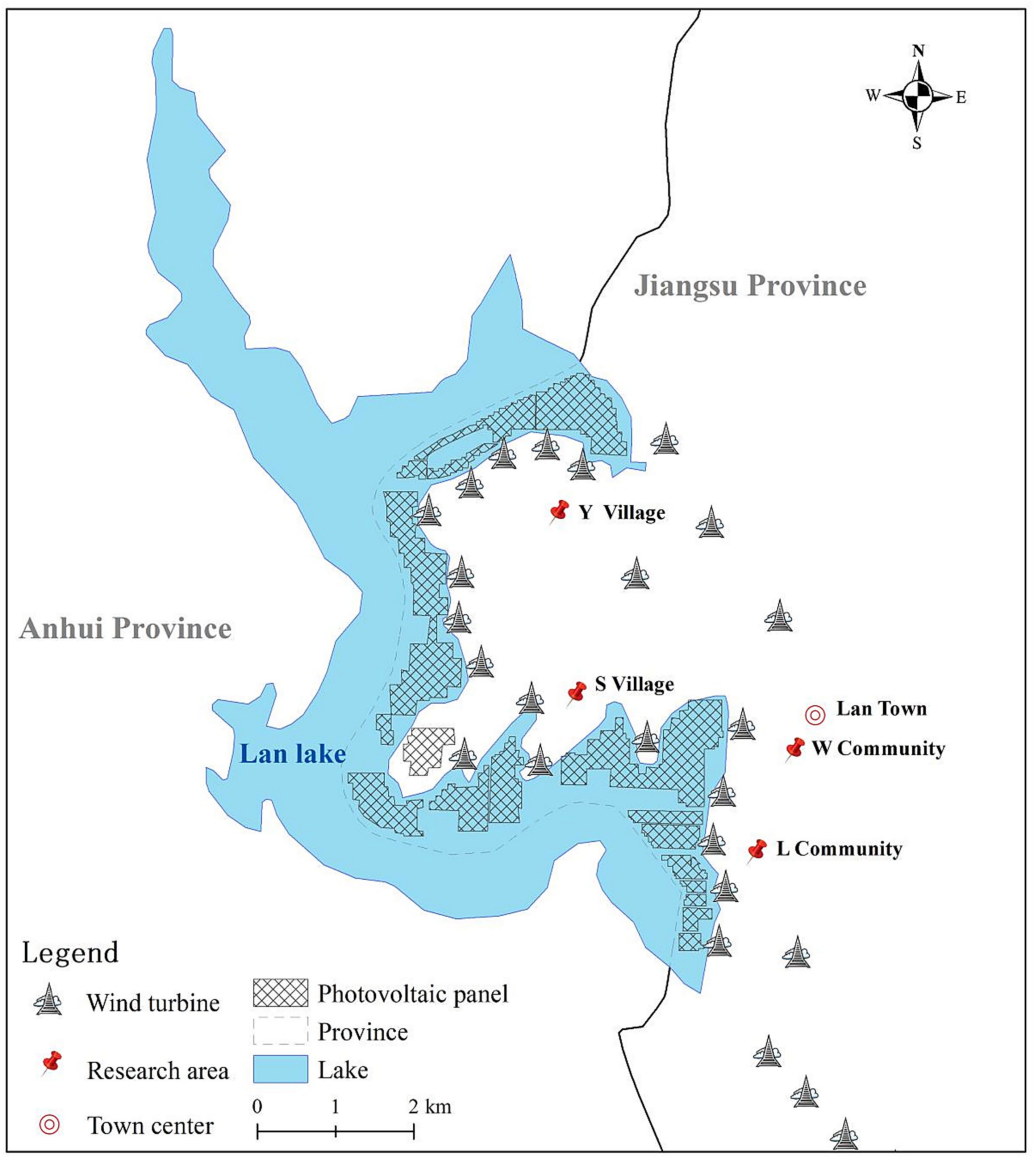
Schematic diagram of the photovoltaic and wind power generation project area in Lan Lake.

The study focuses on the areas most directly affected by these developments. Lan Township as a whole covers 89.18 square kilometers and administers four communities and seven villages. Among these, two communities and two villages were identified as being most affected by the photovoltaic and wind power projects, based on their proximity to the installations. These four areas therefore became the primary study sites (see Figure 4), and all interview and questionnaire participants were recruited from them.

### 3.3 Data collection

Various data collection methods, such as questionnaire surveys, open-ended and semi-structured interviews, and noise measurements, were employed in this study.

#### 3.3.1 Questionnaire survey

We conducted a questionnaire survey in the four areas from October 22 to 25, 2023, and collected 96 resident samples. Purposive sampling as a nonprobability sampling method was employed.^1^ The authors conducted a questionnaire survey in the four areas around Lan Lake mainly affected by the projects: S Village, Y Village, L Community, and W Community. Some people from surrounding communities, villages, or neighboring townships who live, engage in activities, or work in the vicinity of the renewable energy project in Lan Township are included in the “Other” category in Table 1.

**Table 1:** Basic information on the sample.

Item	Category	Number of samples	Percentage (%)
Gender	Male	42	43.75
	Female	54	56.25
Age	20 years old and below	8	8.33
	20–30 years old	11	11.46
	30–40 years old	29	30.21
	40–50 years old	14	14.58
	50–60 years old	14	14.58
	60–70 years old	14	14.58
	70 years old and above	6	6.25
Community	S Village	9	9.38
	Y Village	24	25
	L Community	17	17.71
	W Community	30	31.25
	Other	16	16.67

The male to female gender ratio in the selected samples is 43.75% for males and 56.25% for females. The selected communities and villages are evenly distributed 1–2 km from the coast of Lan Lake, and residents have similar feelings about the photovoltaic and wind projects. The authors selected respondents from different age groups to make the sample as evenly distributed as possible and ensure sampling objectivity (see Table 1). Taking into account the actual employment structure in the local area, the sampled respondents were mainly farmers and part-time fishermen while also including other occupations, such as flexible local employment and people working outside the area, to ensure occupational diversity within the sample.

#### 3.3.2 Interviews

A mixed-method approach was used that combined quantitative and qualitative methods. In addition to collecting data through the questionnaire mentioned above, the authors also obtained research data through field observation and in-depth interviews. Interviews were conducted at three time points: March, June, and October 2023. The authors focused on interviewing key informants to obtain more comprehensive and in-depth information. These informants mainly included staff from the Fishery Management Committee of H County, heads of government departments in Lan Township, staff from photovoltaic enterprises, cadres of community committees, and residents from nearby communities and villages. Open-ended interviews and semi-structured interviews were conducted, with each interview lasting from 30 min to approximately 1–2 h (see Appendix Table 1). In total, this resulted in a valid sample of 22 qualitative interviews.

#### 3.3.3 Noise measurement

To assess noise levels and their impact on residents, the authors used the “Decibel Noise Test” app on an Android mobile phone to measure the noise of wind turbines. The “Map Smart Farming” app was used to test the distance from the wind turbines.

The measurement work was carried out at 5:30 a.m. on June 3, 2023. This time was chosen to capture ambient noise levels from the turbines before sunrise, which is typically a quieter time of day. The measurement location was set at point Y in the residential area closest to the wind turbine in L community, where the population of residents is relatively concentrated. Each point was recorded continuously for at least 5 min to obtain a reliable average noise level value.

### 3.4 Data analysis

After completion of the fieldwork, interview recordings and questionnaire data were analyzed separately and then integrated for interpretation. All interview recordings were transcribed verbatim. Only representative quotations are included in the manuscript to support the findings. Factual information is incorporated into the qualitative descriptions, while statements reflecting residents’ attitudes are presented in the corresponding results subsections.

The interview recordings of the 22 respondents were transcribed and analyzed using “social compatibility” as the overarching concept, and subtopics based on residents’ concerns were identified. In the coding process, based on the residents’ views after the project’s establishment, analysis of the interviews was conducted separately for the photovoltaic and wind energy projects, and qualitative viewpoints were summarized according to three dimensions: environment, resource utilization, and landscape perception. In terms of the environment, the main focus was on the water quality of Lan Lake, the ecosystem, and noise disturbance. Resource utilization and livelihood were mainly operationalized as income, loss of work, and whether power generation brought benefits and increased income. In terms of landscape, this was mainly reflected in perceptions such as whether or not it was considered aesthetically pleasing, among other assessments. Thus, these three dimensions together formed the basis for evaluating social compatibility.

Data were collected via Wenjuanxing,^2^ a professional online survey platform extensively adopted in China for questionnaire distribution, response collection, and preliminary data export. The acquired questionnaire data were then exported into a spreadsheet for subsequent processing. A total of 102 completed questionnaires were received, of which 96 were valid and 6 were invalid. Incomplete responses and samples with logical contradictions were excluded, resulting in a final valid sample of 96 responses. In this study, descriptive statistics, including frequencies and percentages, were employed as the primary approach for quantitative analysis.

After completing the separate analyses, we integrated the two strands of evidence at the interpretation stage. We used the questionnaire results to assess how widely the interview themes were reflects across the larger sample. This triangulation approach strengthened the validity of the study’s conclusions.

## 4 Results

The findings of this study are presented in three subsections. The first subsection examines environmental changes and mutual compatibility, while the second focuses on changes in resource utilization and interest adjustment. The last subsection explains landscape change and mutual adaptation.

Each results section follows a consistent argumentation structure. First, the main findings are presented based on the qualitative research; second, these results are elaborated and compared with the results of the quantitative research; and finally, each section concludes with a summary of the key points.

### 4.1 Environmental change and mutual compatibility

The photovoltaic project has had corresponding environmental impacts on Lan Lake and the local residents. The main environmental impact of the wind energy project on residents is in terms of noise.

#### 4.1.1 Environmental compatibility analysis of the photovoltaic project

The local residents believe that the photovoltaic project has affected the ecological environment of the lake, manifested in changes in three aspects: flood discharge, water quality, and the ecosystem.

First, flood prevention efforts face an increased risk. Since photovoltaic power generation facilities are built on the lake, they occupy the flood channel of the Huai River Basin, which may increase the risk of flood discharge. The normal water level of Lan Lake is only approximately 1 m, but during the rising water period it can reach 2–3 m. The relatively shallow depth of the lake makes it easier to install photovoltaic brackets at the bottom. However, the installation of these brackets affects the flow rate of the lake water. This is especially true when aquatic plants, branches, and other debris float in the water, as they partially block the flood channel. The originally open water area has become narrower, reducing the lake’s flood conveyance and discharge capacity and thereby increasing flood control risks.

Second, the project impacts the water quality of the lake. The development and operation of the project can result in turbidity of the lake water, thereby degrading the water quality. This change has been noticed by local long-term residents, who expressed their views on the deterioration of lake water quality.

*“The river water here is black, and you can see it when you get to the river. The government said it wants clear water and green mountains, but after the photovoltaic panels came in, the river water was not only unclear, but also black. In the past, you could see how deep the bottom of the lake was, but now you can’t. The water in this lake is worse than before. It’s better not to engage in photovoltaic. In the past, the water was clear, and we were often in the lake, and the water was drinkable. Now, the water is ‘Can’t drink.’” [10232023YM1].*

Third, photovoltaic development induces ecological alterations via changes to the aquatic environment. Although installed at an inclined angle, photovoltaic panels still cover extensive areas of the lake surface. Long-term underwater light deprivation constitutes a major ecological stressor. By absorbing and reflecting solar radiation, the panels restrict light penetration into deep water layers, inhibiting the growth of aquatic vegetation and reducing overall plant biomass. Such light limitation also exerts microscopic influences on zooplankton and microbial communities, thereby indirectly altering the inherent structure of the aquatic ecosystem.

*“There are photovoltaic panels on top, but there is no light on the bottom. The aquatic plants are basically gone and no longer grow. The lack of sunlight has some effect.” [10232023SM3].*

The person in charge of the fishery management department of Lan Township government agreed with the concern about environmental change.

*“Some people say there is no impact, but photovoltaics use sunlight, and the lack of sunlight on the water surface must be affected to some extent, and plankton and zooplankton will definitely use light.” [0302023WZR].*

This is particularly evident in reduced aquaculture production. Photovoltaic panel coverage constrains fish growth and impedes local fishery operations to a certain degree. This effect primarily stems from sunlight obstruction, which restricts normal fish development and subsequently lowers overall yield. According to local aquaculture personnel’s estimations, aquaculture under photovoltaic arrays yields approximately 50% less than conventional lake farming. Both captive fish stocks and the natural habitats of wild fish are substantially affected.

*“Photovoltaics have an impact on fish. If this board is placed on top and there is no sunlight underneath, its growth will be poor. As long as there is sunlight, of course the fish will grow well.” [10232023SM3].*

A member of the power station staff from a photovoltaic enterprise also noted the following:

*“This project is still based on photovoltaics, and the fish stocking density may have to be reduced by half.” [0302023WZR].*

**Figure 5:**
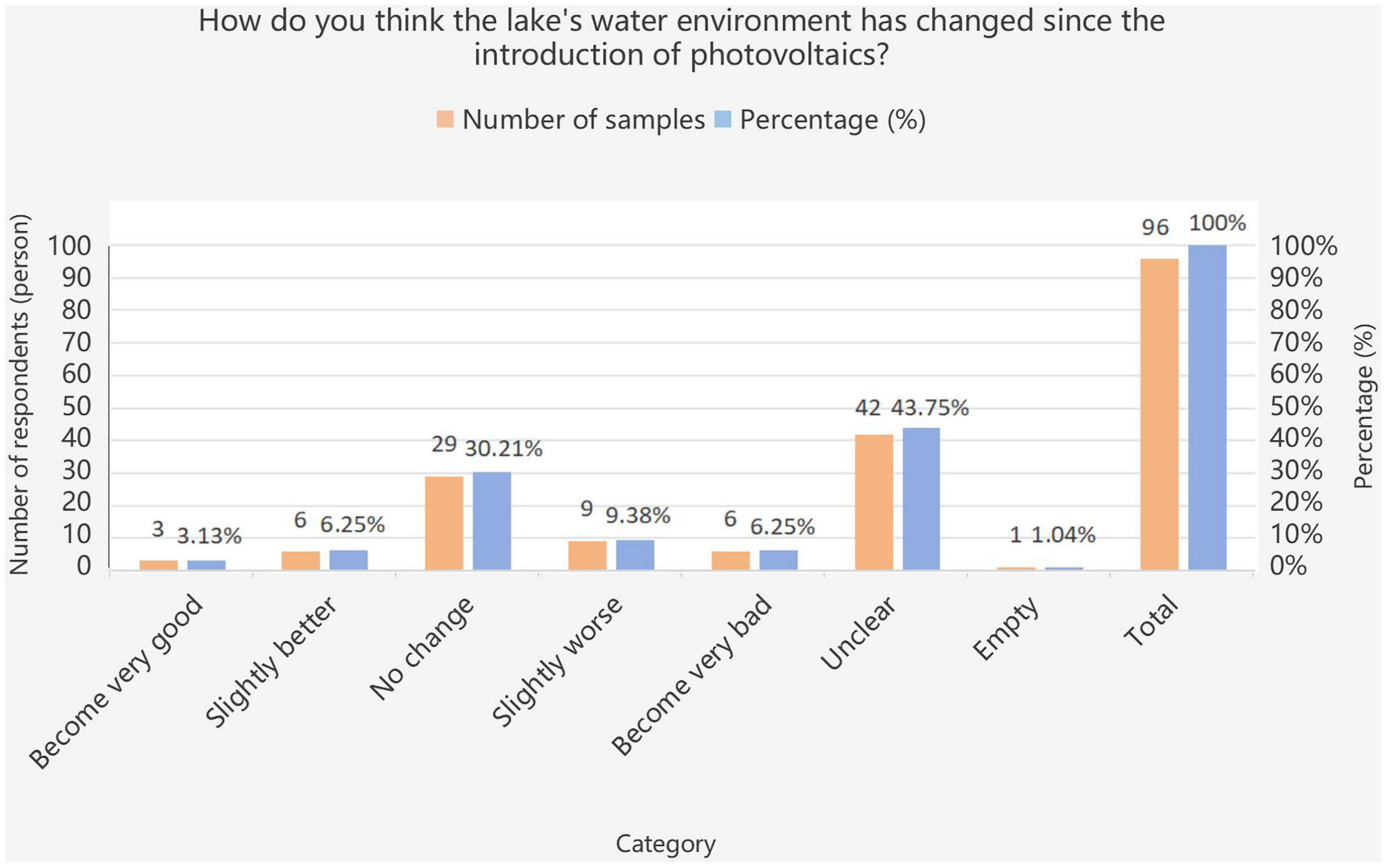
Extent of photovoltaic impacts on the lake’s water environment. Source: Field survey. Water environment refers to the spatial environment in which water is formed, distributed, and transformed in nature. The water environment in this paper mainly refers to the water quality of Lan Lake.

The quantitative results indicate that the photovoltaic project’s impact on the local water environment has raised residents’ concerns. Figure 5 illustrates residents’ perceptions of the degree of this impact. Some residents expressed that the impact is unclear, possibly because photovoltaic projects involve complex technical issues that ordinary residents do not examine in depth. The results show that 30.21% of residents believed that the lake’s water quality had not changed significantly. The proportions of those who felt it had become slightly better and slightly worse were 6.25 and 9.38% respectively, while 6.25% considered it to have become very bad. Overall, a portion of residents reported that the water environment of Lan Lake had become worse than before. When explaining the reasons, residents made comments such as “the water has become dirty,” “the water in the lake is black,” and “it has become stagnant.”

#### 4.1.2 Noise compatibility of the wind energy project

Wind turbines generate continuous noise during operation, which was a recurring concern among residents living near the project. According to Chinese national standards (GB3096-2008, GB12348-2008), noise from wind turbines should not exceed 60 dB during the day and 50 dB at night. The minimum distance between a turbine and residential areas is recommended to be 200 m. Field measurements conducted by the authors at 5:30 a.m. using a mobile phone–based noise meter showed that sound levels at distances of 180, 210, and 240 m from the turbines were all 50 dB. The nearest residential house was approximately 180 m from a turbine, and the distance from the center of the residential area was about 200 m. According to Table 2, these measurements indicate that the project complies with national noise standards.

**Table 2:** Distribution of noise level and wind turbine distance in L community.

Distance to wind turbine (m)	Average decibel every 5 min (dB)	The feeling of noise
0 m	62	Very noisy, similar to the roar of an airplane
50 m	54	Very noisy, similar to the hum of a water-jet loom in a factory
100 m	50	Noisy, similar to the buzzing sound of the external unit of the air conditioner
180 m (edge of residential area)	50	Noisy, similar to the buzzing sound of the external unit of the air conditioner
210 m (residential center)	50	Noisy, similar to the buzzing sound of the external unit of the air conditioner
240 m (edge of residential area)	50	Noisy, similar to the buzzing sound of the external unit of the air conditioner

Source: Field survey.

Despite meeting legal requirements, noise remained a source of dissatisfaction among residents. One resident living near a turbine described the sound: “It buzzes like an airplane when I sleep at night” [10222023SM2]. Another stated: “The sound of the wind turbine is whining. Sometimes I hate it. When I sleep at night, if the doors and windows are not good, it keeps me awake” [10242023LM1]. A community committee member confirmed that noise could be heard up to 1 km away [06032023MZR]. Local farmers also reported that they could not sleep at noon, and that closing windows did little to reduce the noise, especially in the early morning and evening hours.

In Table 3, the questionnaire results further quantified the extent of noise-related discomfort. Among the 96 respondents, 39.58% reported no noise impact, while the remaining about 60.42% reported some level of impact or uncertainty: 21.88% perceived a moderate impact, 19.79% a major impact, and 15.63% a minor impact, and 3.13% were unsure. No respondent selected “extraordinary impact.”

**Table 3:** Noise impact of wind turbines on residents.

How do you think the noise impact has been since the wind project came in?	Number of samples	Percentage (%)
Very influential	0	0
Have a greater impact	19	19.79
General impact	21	21.88
Have minor impact	15	15.63
No impact at all	38	39.58
Unclear	3	3.13
Total	96	100

Source: Field survey.

The variation in perceived impact appears to be explained largely by spatial distance. Residents living closer to the turbines consistently reported stronger discomfort, while those farther away were less affected. As one resident noted: “The people who are close find it noisy, but it does not bother us if we are far away” [10222023SF3]. This pattern suggests that even when noise levels fall within legal limits, proximity matters for residents’ subjective experience.

#### 4.1.3 Summary

The photovoltaic project, built on a lake, primarily affects the water environment. While its technical impacts are difficult to verify, residents have raised legitimate concerns based on changes in flood discharge, water quality, and the ecosystem. In contrast, the wind energy project mainly generates noise, which has led to clear incompatibility with nearby residential areas. Overall, the qualitative findings presented in this section are consistent with the quantitative results.

### 4.2 Changes to resource utilization and livelihoods

The development of renewable energy projects has led to changes in resource utilization methods and the distribution of benefits. Specifically, it has resulted in a decline in the income of the fishing community, the substitution of their means of livelihood, and an unequal distribution of benefits among the residents in the region.

#### 4.2.1 Livelihood changes of fishermen and land-based farmers

The change in resource utilization has caused most fishermen to lose their source of fishery income. Before the project was officially launched, residents living in the surrounding areas could benefit from the resource advantages of the lake. For fishermen operating on an average water surface of 1.33–2 ha, their annual income from breeding is 20,000–30,000 yuan/year. Some fishermen with good harvests have 1.33 hectares of water surface and earn 70,000–80,000 yuan a year. Some large fishermen have a water surface area of 4.67 ha, which can generate an annual income of 200,000–300,000 yuan.

Similar to land property rights, the water surface belongs to the state and is a public good; however, in traditional practices, fishermen obtaining the right to use the water surface is tacitly tolerated. When the project began, the right to use the water surface was reclaimed by the state for the development of renewable energy projects. Fishermen lost the right to fish and practice aquaculture on the surface. After the installation of photovoltaic projects, fishermen can no longer derive any income from fisheries.

As Lan Lake is now occupied by the project, farmers can no longer freely access the water surface for fishing, whereas previously they used to be able to go to the lake to fish and catch shrimp. The per capita land in Lan Township is 0.1–0.13 ha, while a family of four to five people has about 0.4–0.67 ha of land. Landed farmers lost their fishing livelihoods, reducing their income from surface fishing. If they earned an average of 50 yuan a day from selling fish and shrimp and went fishing in the lake 20 days a month, they could earn 1,000 yuan a month. This is a considerable income for farmers who mainly farm land. In the sample in Table 4, the proportion of land-owning farmers before the project began was 36.46%, accounting for most local residents. Therefore, the impact on farmers cannot be ignored. Some local farmers brought up this issue in the interviews:

**Table 4:** Occupations of residents before and after the photovoltaic project was established.

Occupation type	The number of samples before entering the project	The number of samples after entering the project	Percentage (%) increase or decrease
Aquaculture farmer	1	2	100
Farmer	35	36	2.86
Part-time fisherman	18	3	−83.33
Engineering contracting	1	1	0
Temporary workers around	2	3	50
Migrant workers	27	7	−74.07
Nearby employment	9	11	22.22
Student	7	7	0
Unemployed	11	21	90.91
Other	17	23	35.29
Total	128	114	−10.94

Source: Field survey. Other occupations mainly include village or community cadres, self-employed individuals, freelancers, etc.

*“In the past, farmers could go to the lake to catch some fish and shrimps. Some could sell them for forty or fifty yuan a day, and some could earn one or two hundred, or even three or four hundred. After photovoltaics came in, we could not make any money.” [10232023SM3].*

*“Now we have no income from fishing fish and shrimp, so we can only plant some land.” [10222023SF3].*

*“We are older adults and cannot find jobs when we go out.” [10222023SM2].*

Changes in resource use have resulted in fisher groups losing their livelihoods and professional fishermen being forced out of the waters. There were originally more than 500 aquaculture households in Lan Township. After the photovoltaic project began, only a few people remained. As of October 2023, about 50 or 60 such households were left in Lan Township, and they could only engage in aquaculture on certain tidal flats or in ponds. Only two companies can engage in fishery farming under photovoltaic panels. One of them is independently operated by W photovoltaic enterprise, while another photovoltaic company contracted the water surface to a fishing company for operation. They established a cooperative to share dividends, but only a little over 10 aquaculture households participated. Most residents can no longer participate in fishing and aquaculture. A local farmer engaged in fisheries said:

*“The impact on the fishermen has been great. They have all lost their jobs. You see, we do not have much land in the surrounding villages, so we just hope to make a living by doing fish farming in the lake. They expropriated the lake; the people will have no choice but to go out to work.” [03012023LXS].*

There have also been changes in residents’ occupations and incomes, as verified statistically in the quantitative results. As shown in Table 4, among the occupational changes of residents after the project was established, part-time fishermen largely disappeared. After the fishermen lost their livelihoods, some were unemployed, and some turned to other jobs. Most older adults who had been part-time fishermen and farmers continue to engage in agriculture and are less likely to take non-local employment opportunities. However, the majority of those engaged in fishing are middle-aged and older adults. As a result, the number of unemployed people increased significantly, while the number of farmers, nearby employees, migrant workers, and other occupations account for a large proportion of the total workforce (Table 4).

Table 5 shows the impact on residents’ income after the project was established. A small number of residents, accounting for 3.13% of the total, have experienced a significant loss of income due to the photovoltaic project. These individuals are mainly professional fishermen and part-time fishermen engaged in fisheries. The proportion of residents whose income has decreased slightly account for 16.67% of the total, mainly surrounding farmers engaged in natural fishing. For other ordinary residents, the impact has been relatively small. The proportion of residents whose income remained basically unchanged account for 70.83%. For residents who do not rely on fishery resources for their livelihood, the photovoltaic project has not directly affected their income sources and career choices.

**Table 5:** Changes in residents’ income after the photovoltaic project was established.

Changes in income	Number of samples	Percentage (%)
Improve a lot (more than 60%)	2	2.08
Improve some (within 20–60%)	4	4.17
Basically, no change (−20–20%)	68	70.83
Reduce some (within 20–60%)	16	16.67
Reduce a lot (more than 60%)	3	3.13
Empty/do not know	3	3.13
Total	96	100

Source: Field survey.

#### 4.2.2 Inequality and incompatibility of benefit distribution

After the project was established, it theoretically led to local economic development; however, ordinary residents have benefited very little from it. After the photovoltaic project began operating, enterprises were able to obtain high revenues from electricity sales. Based on an electricity price of 0.37 yuan/kWh and a subsidy of 0.96 yuan/kWh, the income from electricity sales is approximately 1.33 yuan/kWh. W Enterprise’s total annual power generation is 140 million kilowatt-hours. Excluding losses, the grid-connected electricity is 130 million kilowatt-hours per year, and its annual output value can reach 170 million yuan. Compared with the high output value and high profits of the photovoltaic project, fishermen’s compensation benefits are negligible. For example, a household with 1.33 ha of water surface only receives a compensation income of 18,000 yuan. Some local farmers who are not engaged in fishing also said:

*“It is good for the country and the government to develop renewable energy, and we certainly agree with it, but it is not good for farmers.” [10222023SM1].*

*“These projects occupy our land and water surface, and we can’t accept them. There is no reduction or discount on electricity charges; their benefits have nothing to do with us.” [1022023SF1].*

The local government of Lan Township generates 70 million yuan in annual tax revenue for the county government through photovoltaic projects. There are a total of six photovoltaic enterprises in Lan Lake, and the tax revenue of three of them belongs to the Lan Township government. The total output value of these three enterprises is about 200 million yuan, and the tax revenue is approximately 30–40-million-yuan, accounting for 44% of the total tax revenue of the township.^3^

Economic growth has not led to the direct distribution of benefits among the residents. With the arrival of the project, some public infrastructure in market towns and streets could be built. The project was able to provide some employment opportunities for local residents in the early stage of construction, mainly heavy manual labor and ancillary positions. After the project became operational, fishermen, who generally had low educational levels, found that their employment skills did not match those of the enterprises. In addition, the photovoltaic enterprises themselves employed less labor, so they could provide few suitable jobs. An accountant in Y Village stated that there are three photovoltaic companies in their village, which employ no more than five laborers from the village in total [03012023YKJ]. Overall, the establishment of photovoltaic projects has changed the resource utilization methods of local residents who rely on fisheries for their livelihoods, making it difficult to resolve the incompatibility between residents and the project through factors such as compensation and employment.

#### 4.2.3 Summary

For resource utilization compatibility, local fishing communities bear the most direct impacts on their livelihoods. Their income declines as traditional livelihood channels are restricted, forcing them to seek alternative occupations. Although the projects generate certain benefits, these gains are not shared with local residents, resulting in unequal economic distribution. Qualitative and quantitative results are highly consistent, demonstrating economic incompatibility between project development and household income, as local residents are excluded from benefit distribution.

### 4.3 Landscape change and mutual compatibility

Residents’ views on the landscape vary between project types. This section therefore presents their opinions on photovoltaic and wind energy landscapes separately.

#### 4.3.1 Landscape compatibility of the photovoltaic project

This section analyzes the quantitative and qualitative results, which indicate that residents perceive the landscape compatibility of the photovoltaic project as relatively poor.

It has been noted that landscape changes resulting from the transition to renewable energy are one of the main objections of local stakeholders (32). Photovoltaic panels are the main devices used to collect solar energy and convert it into electricity. Regarding water surface, photovoltaic panels cover nearly 70% of the lake surface in Lan Township, creating a new steel-framed architectural space on the water.

The subjective cognition of landscape aesthetics is manifested in four aspects: perception, imagination, understanding, and emotion (33). Landscapes can be seen as an emotional creation (34). A sense of place attachment has been highlighted as a critical factor in the social acceptance of local communities (35). Local residents in Lan Township have also expressed consistent objections to landscape changes caused by photovoltaic projects, which are mainly reflected in their negative perceptions of photovoltaic panel coverage.

Some residents do not perceive the photovoltaic landscape as aesthetically pleasing and hold negative attitudes toward it. They prefer the original natural landscape, which they believe has been destroyed by the photovoltaic project. Combined with the project’s negative impacts on their lives and livelihoods, these residents have developed a sense of rejection. For example, Mr. Wang, owner of a hotel in Community W and a former community committee cadre, stated:

*“I don’t like to look at photovoltaic panels. It destroys the original ecology. At first, there were seine nets in the lake and there were reeds on the shore. Isn’t this the original ecology?” [06032023WZR].*

*“I definitely thought it looked good when I first watched it. At first, I didn’t know much about photovoltaic panels, but now I feel it’s better to look at the lake.” [10242023WZR].*

Other residents had similar views and expressions.

*“I like the original ecology. We are used to it. It looks the same as before. After a long time, we can no longer feel the characteristics of this photovoltaic. The lake surface used to be so beautiful, but now it is covered.” [06032023HZR].*

*“What is there to see? Nothing but photovoltaic panels.” [10222023SM1].*

*“I don’t think it’s beautiful. I feel like there’s not much greening since the photovoltaic came in. In the past, the natural landscape in our lake was pretty good.” [10222023SF1].*

Compared with the qualitative findings, the quantitative results enable the classification of different viewpoints. The results show that less than a third of residents in the sample like Mr. Wang shared negative attitudes, accounting for 26.04% of the total. Among them, 23.96% viewed it as unsightly, while 2.08% perceived it as very unsightly. The questionnaire survey revealed that, overall, residents’ evaluations of the landscape were more “neutral” and “balanced.” In Table 6, the second category of residents had the highest proportion of “neutral” attitudes. They expressed “no feeling” about the photovoltaic landscape, accounting for 35.42% of the total. This category of residents accounts for slightly more than a third of the sample. They neither perceived the photovoltaic project as beautiful nor thought it ugly. Some residents pointed out that “photovoltaic” has little to do with “beauty.” Some residents also noted that they have no feelings about the photovoltaic landscape because they are more concerned with their livelihoods; whether it is beautiful or not does not play a significant role in the lives of local people. However, when asked for an overall assessment, 41.67% of respondents deemed the photovoltaic project incompatible (see Appendix Table 2).

**Table 6:** Residents’ feelings about the beauty of photovoltaic landscape.

Do you think the landscape of photovoltaic projects is beautiful?	Number of samples	Percentage (%)
Very beautiful	3	3.13
More beautiful	25	26.04
No feeling	34	35.42
Unsightly	23	23.96
Very unsightly	2	2.08
Unable to evaluate	9	9.38
Total	96	100

Source: Field survey.

The third category of residents held a relatively positive attitude toward the photovoltaic landscape: 26.04% found it relatively beautiful and 3.13% very beautiful, accounting for 29.17% of the total. Thus, the proportion of residents who perceived the photovoltaic landscape as beautiful is also close to a third of the sample, which is consistent with some residents’ views that they do not reject the photovoltaic landscape and even recognize its aesthetic value. In addition, 9.38% of the sample were “unable to evaluate” whether photovoltaics is beautiful or not, indicating that some people also find photovoltaics difficult to judge from an aesthetic point of view. Overall, residents’ positive, neutral, and negative evaluations all account for approximately a third of all responses, as shown in Table 6.

Residents’ perceptions of the project’s landscape are not fixed. Some residents said they were more receptive to the photovoltaic project when it was first implemented. As they obtained a more in-depth understanding of photovoltaic projects, they slowly found it less beautiful. Some residents also said that as time went by, their discomfort weakened, and they gradually got used to it. Residents’ evaluations of the project’s landscape changes with their needs and emotions and cannot be reduced to a simple binary opposition between “beautiful” and “ugly.”

#### 4.3.2 Landscape compatibility of the wind energy project

Compared with residents’ mixed perceptions of photovoltaic landscapes, their attitudes toward wind turbine landscapes show clear differences, with a higher level of acceptance.

Wind power projects require the construction of a number of wind turbines near communities or villages, and these turbines also form an important part of the landscape. The wind turbines in Lan Township are mainly distributed along the lake shore, with some scattered across the village fields. Different residents have different views on this landscape. This article classifies residents’ opinions into three categories: positive, negative, and neutral.

First, 53.12% of the residents expressed positive attitudes toward the wind turbine landscape. As shown in Table 7, the perception “relatively beautiful” accounts for 45.83% and “very beautiful” accounts for 7.29% of the total. This shows that these residents can appreciate the beauty of wind turbines and recognize their landscape value. For example, in terms of shape, wind turbines have a modern aesthetic, and some cities imitate the shape of wind turbines as an urban landscape element to enhance the aesthetics of urban space. As an industrial landscape, wind turbines take into account modern aesthetics, which contributes to greater acceptance among residents.

**Table 7:** Residents’ feelings about the beauty of wind turbine landscapes.

Do you think wind turbines are beautiful?	Number of samples	Percentage (%)
Very beautiful	7	7.29
More beautiful	44	45.83
No feeling	28	29.17
Unsightly	12	12.5
Very unsightly	0	0
Unable to evaluate	5	5.21
Total	96	100

Source: Field survey.

Second, 12.5% of the residents expressed negative attitudes toward the beauty of the landscape, but this proportion is not high (see Table 7). This group of residents clearly expressed their disapproval of the landscape value of wind turbines by stating that they were “unsightly.” Some residents even had some resentment toward wind power projects due to the noise, while others believed that wind turbines are an obstacle on the landscape. They are installed in various places in communities and villages and cannot provide people with aesthetic enjoyment. Not only that, but the rotation of wind turbines also invisibly gives people a sense of anxiety and oppression.

*“It looks pretty good, but I feel irritated and the noise is too loud.” [10242023SZR].*

*“When you see a windmill for the first time, the noise is nothing. But if you live here every day, you will feel irritated. Normal people who live here will not have a good impression of wind turbines.” [10232023YM1].*

Third, about one-third of the residents expressed neutral attitudes toward this issue. Specifically, 29.17% reported having no feeling about wind turbine landscapes. Attitudes such as “I do not think it looks good or not” and “I’ve gotten used to it after living there for a long time; it does not matter whether it looks good or not” indicate that they have indifferent views toward the landscape of wind power projects. The residents who were unable to offer an evaluation accounted for 5.21% of the total. They expressed the following views: “I do not know whether it looks good or not” and “cannot answer.” Therefore, a small number of residents said that it was difficult to evaluate the aesthetics of the landscape. Classifying “no feeling” and “unable to evaluate” into one category shows that residents have no clear preference regarding the landscape of wind power projects. This group accounted for 34.38% of residents.

In general, residents showed higher acceptance of the aesthetics of wind turbines than of photovoltaic landscapes. In the above data, the combined proportion of residents who perceived the photovoltaic landscape as “relatively beautiful” and “very beautiful” is 29.17%, while corresponding proportion for the wind turbine landscape is 53.12%. The high acceptance rate of wind turbine landscapes is also reflected in residents’ descriptions of their modern aesthetic characteristics. Overall, more than half of the residents expressed approval of the landscape of wind power projects.

#### 4.3.3 Summary

Regarding landscape compatibility, the results reveal widespread perceived incompatibility between energy projects and local landscapes. Moreover, distinct differences exist between photovoltaic and wind energy developments: photovoltaic installations exhibit notably lower landscape compatibility than wind projects. Nearly one-third of residents held negative attitudes toward photovoltaic landscapes, which aligns with qualitative interview evidence showing widespread dissatisfaction regarding damage to original natural scenery.

Two key implications emerge from these findings. First, landscape compatibility is not a binary construct, as many respondents maintained neutral or ambivalent perceptions. Second, compatibility varies substantially across energy technologies: photovoltaic projects encounter stronger landscape-related opposition than wind energy facilities. Accordingly, future siting and design strategies should be technology-specific, with enhanced emphasis on visual integration for photovoltaic infrastructure.

## 5 Discussion

This study, based on the case of Lan Lake in the Huai River Basin, has explored the social compatibility of photovoltaic and wind energy projects in rural Eastern China, revealing key findings across environmental, livelihood, and landscape dimensions. This section interprets these results in the context of existing literature, highlights the study’s contributions, and discusses their theoretical and practical implications.

The findings show that the installation of the photovoltaic project in the Lan Lake area has exerted negative environmental impacts, particularly posing significant risks to flood control, a concern also recognized by the local Water Conservation Authority. This aligns with national policy requirements: the Ministry of Water Resources (36) explicitly prohibits the construction of renewable energy projects (including photovoltaic and wind energy) in rivers, lakes, and reservoirs in its Guiding Opinions on Strengthening the Spatial Management and Control of Coastlines of Rivers and Lakes, classifying such installations as illegal structures. The lack of consideration of environmental impacts in the photovoltaic project’s initial design not only violates relevant policies but also undermines ecological security, highlighting the need for stricter preproject environmental assessments.

The environmental impacts of photovoltaic panels and wind energy turbines further illustrate the complexity of renewable energy development. Local communities reported mixed perceptions of changes in water quality, characterized by increased turbidity and stagnation. While the wind turbines closest to residential areas meet noise standards, the noise generated has adversely affected residents’ quality of life and sleep. These findings support and extend those of previous research Grippo et al. (46) review the potential impacts of solar energy development on aquatic habitat and biota. Exley et al. (26) and Lafitte et al. (37) confirmed that solar panels can alter aquatic ecosystems by reducing water temperature fluctuations and light penetration, which is consistent with the observations in this study of photovoltaic-related water quality changes. Similarly, Jensen et al. (38) and Nazir et al. (39) documented the social impacts of wind turbine noise, particularly its disturbance to surrounding communities, corroborating our findings on residents’ sleep and quality of life. Notably, most existing studies have focused on the technical aspects of renewable energy’s environmental impacts, with less attention paid to policy compliance and social perceptions (e.g., ecological risk prevention and community attitudes). This study addresses this gap by integrating environmental, policy, and social perspectives.

The introduction of photovoltaic projects has also significantly transformed residents’ livelihoods, disproportionately affecting farmers and fishermen who rely on Lan Lake for their income. While the projects have generated certain economic benefits, ordinary residents have not benefited from these gains. This finding is consistent with studies conducted in other geographic contexts: Erdiwansyah et al. (40) and Nguyen et al. (41), in their research on renewable energy projects in Southeast Asia, emphasized the often-overlooked social costs of resource-intensive renewable energy development. From the perspective of economic equity, this study highlights the unfair distribution of project benefits, which has led to interest infringement and economic losses for some groups, triggering social injustice disputes (e.g., conflicts over land and property rights, and declines in income and quality of life). These findings underscore the importance of integrating equity considerations into renewable energy project planning.

Furthermore, renewable energy developments have significantly altered the local landscape, eliciting diverse community responses: some residents hold neutral or positive attitudes toward the new industrial landscape, while others mourn the loss of the original natural landscape. This aligns with Hallan et al. (47) Enserink et al. (32) and Prados et al. (35), who observed varied community responses to environmental changes caused by renewable energy installations. However, existing studies have primarily focused on the visual barriers of such projects. For example, Gibbons (42) assessed the visual impact of wind turbines through changes in house prices, and Devine-Wright and Batel (43) noted that new power lines often spark controversy due to their negative impact on rural landscapes. This study extends this line of research by focusing on landscape aesthetics, exploring how rural communities perceive changes in their visual environment. The findings highlight the critical role of landscape strategies in fostering public acceptance of renewable energy projects and enrich understanding of community perspectives on environmental changes in rural contexts.

Theoretically, this study enriches the literature on the social and environmental costs associated with renewable energy development. It advocates a balanced approach to environmental impact assessment that integrates ecological protection and human well-being concerns, thereby clarifying the tensions between climate mitigation goals and socioeconomic equity at the local level. By examining rural residents’ perceptions of changes in the environment, livelihoods, and landscapes throughout the energy transition, this study advances understanding of the socioeconomic impacts of renewable energy development, especially in developing countries, and highlights the importance of incorporating diverse social dimensions into sustainable development frameworks.

### 5.1 Conclusion and policy implications

This study explored renewable energy project development from the perspective of social compatibility, which complements the existing research focus on technical and economic benefits and provides a new perspective for the sustainable development of renewable energy.

The research examined the social compatibility of photovoltaic and wind energy projects with rural communities in Eastern China, using a mixed-methods approach that combined semistructured interviews and a questionnaire survey conducted in four villages surrounding existing renewable energy installations. By focusing on residents’ perceptions of environmental change, land and resource use, and landscape aesthetics, the study aimed to identify sources of compatibility and incompatibility between renewable energy development and local communities.

Three main findings emerged. First, environmental perceptions were mixed. Although some residents acknowledged the potential environmental benefits of renewable energy, concerns were raised regarding local ecological impacts, particularly perceived declines in water quality and fish harvests adjacent to photovoltaic installations. Second, land and resource use emerged as a source of tension. The transition from traditional fishing and farming to renewable energy operations was associated with reported job losses and income reductions, with the questionnaire data indicating that the perceived economic benefits remained limited. Third, landscape compatibility was the most salient dimension of incompatibility. Although wind turbines were relatively accepted by residents, photovoltaic projects faced stronger opposition regarding their visual integration, with about one-third of survey respondents expressing negative perceptions.

Based on these findings, three policy strategies are proposed. First, participatory planning mechanisms should be established to incorporate community input into project siting and design, particularly given the centrality of residents’ landscape concerns. Second, livelihood transition support, including skills training and revenue-sharing mechanisms, is needed to address economic disruptions beyond standard compensation schemes. Third, differentiated approaches by technology type are warranted, as photovoltaic and wind energy projects generate different patterns of social response.

Several limitations of the study should be acknowledged, including the regional specificity of the sample, the cross-sectional design, and the convenience sampling approach. Future research should extend this work through multiregional comparisons, longitudinal designs, and intervention studies that evaluate the effectiveness of participatory planning mechanisms. Such efforts will contribute to developing evidence-based guidelines for socially compatible renewable energy transitions.

## Data Availability

The original contributions presented in the study are included in the article/Supplementary material, further inquiries can be directed to the corresponding author.

## Footnotes

1 Purposive sampling, also known as judgment sampling, is a type of nonprobability sampling in which observation objects are selected based on personal judgment (whether the object is valid or most representative).

2
www.wjx.cn

3 The data are based on interviews with staff from the Lan Township Agriculture and Rural Affairs Office.
