# Supplementary material for: Is the development of renewable energy projects compatible with rural communities? The case of Eastern China
Source: Front Public Health. 2026 May 15;14:1789344. doi: 10.3389/fpubh.2026.1789344 (PMC13219018; doi:10.3389/fpubh.2026.1789344)
Supplement: Supplementary file 1 [file Data_Sheet_1.docx]

**Appendix Table 1.** List of main interviewees.

| Interview time | Interviewee and code | Interview location | Interview duration |
| --- | --- | --- | --- |
| March 1, 2023 | Director Shen, Agriculture and Rural Affairs Office of Lan Township Government [03012023SZR] | Government department office | 50min |
| March 1, 2023 | Retired Secretary Zhao, Z Community Management Committee of Lan Township [03012023ZSJ] | Community Management Committee Office | 40min |
| March 1, 2023 | Accountant Yao, Y Village Committee of Lan Township [03012023YKJ] | Village committee office | 30min |
| March 1, 2023 | Mr. Li, an individual aquaculture farmer in S Village of Lan Township [03012023LXS] | Near Lan Lake Photovoltaic Project | 38min |
| March 2, 2023 | Director Wang, Fishery Management Department of Lan Township [0302023WZR] | Government department office | 2h |
| March 2, 2023 | Mr. Zhou, a staff member of the photovoltaic power station of W enterprise in Lan Township [03022023ZXS] | Photovoltaic Project Office |  |
| March 3, 2023 | Director Zhang, Industrial Office of Lan Township Government [03032023ZZR] | Government department office | 43min |
| June 1, 2023 | Director Wei, Fishery Management Committee of H County Government [06012023WZR] | Government department office | 2h27min |
| June 2, 2023 | Director Yan, Agriculture and Rural Affairs Office of Lan Township Government [06022023YZR] | Government department office | 1h22min |
| June 3, 2023 | Retired Director Wang, W Community of Lan Township [06032023WZR] | Lan Township Hotel | 38min |
| June 3, 2023 | Director Ma, L Community Management Committee of Lan Township [06032023MZR] | Community Management Committee Office | 43min |
| June 3, 2023 | Director Hua, S Village Committee of Lan Township [06032023HZR] | Village committee office | 53min |
| October 22, 2023 | Middle-aged female in S Village, Lan Township [10222023SF1] | Village | 32min |
| October 22, 2023 | Middle-aged female in S Village, Lan Township [10222023SF2] | Village | 40min |
| October 22, 2023 | Middle-aged male in S Village, Lan Township [10222023SM1] | Village | 43min |
| October 22, 2023 | Older adult male in S Village, Lan Township [10222023SM2] | Village | 20–30min |
| October 23, 2023 | Older adult female in S Village, Lan Township [10222023SF3] | Village | 19min |
| October 23, 2023 | Older adult male in S Village, Lan Township [10232023SM3] | Village | 20–30min |
| October 23, 2023 | Middle-aged male in Y Village, Lan Township [10232023YM1] | Village | 30min |
| October 23, 2023 | Middle-aged female in Y Village, Lan Township [10232023YF1] | Village | 20min |
| October 24, 2023 | Older adult male in Y village, Lan Township [10242023YM2] | Village | 20min |
| October 24, 2023 | Older adult male in L community, Lan Township [10242023LM1] | Community | 24min |
| October 25, 2023 | Older adult male in W Community, Lan Township [10252023WM1] | Community | 25min |

**Appendix Table 2.** Residents’ overall compatibility evaluation of photovoltaic and wind power projects.

| Do you think this project is suitable? | Photovoltaic project | Percentage (%) | Wind power project | Percentage (%) |
| --- | --- | --- | --- | --- |
| very suitable | 5 | 5.21 | 7 | 7.29 |
| More suitable | 15 | 15.63 | 17 | 17.71 |
| generally | 26 | 27.08 | 34 | 35.42 |
| unsuitable | 33 | 34.38 | 26 | 27.08 |
| Very unsuitable | 7 | 7.29 | 4 | 4.17 |
| Unable to evaluate | 10 | 10.42 | 8 | 8.33 |
| total | 96 | 100 | 96 | 100 |
